# Draft Genome Sequence and Annotation of Paracoccus versutus MAL 1HM19, a Nitrate-Reducing, Sulfide-Oxidizing Bacterium

**DOI:** 10.1128/MRA.01419-19

**Published:** 2020-03-05

**Authors:** Wannapawn Watsuntorn, Thammaporn Kojonna, Eldon R. Rene, Piet N. L. Lens, Warawut Chulalaksananukul

**Affiliations:** a Department of Botany, Faculty of Science, Chulalongkorn University, Bangkok, Thailand; b Biofuels by Biocatalysts Research Unit, Faculty of Science, Chulalongkorn University, Bangkok, Thailand; c Center of Excellence in Environment and Plant Physiology, Faculty of Science, Chulalongkorn University, Bangkok, Thailand; d UNESCO-IHE Institute of Water Education, Delft, The Netherlands; University of Maryland School of Medicine

## Abstract

Paracoccus versutus MAL 1HM19 is a mixotrophic nitrate-reducing sulfide-oxidizing bacterium which plays a crucial role in hydrogen sulfide (H_2_S) and nitrate (NO_3_^−^) removal. In this study, we report the draft genome sequence of *P. versutus* MAL 1HM19.

## ANNOUNCEMENT

The genus *Paracoccus*, phylogenetically classified in the class *Alphaproteobacteria*, comprises Gram-negative, nonmotile bacteria which belong to the facultative chemolithotrophic sulfide-oxidizing bacteria (1). They have been isolated from many sources, including bioreactors, sludge, aerobic granules, sediments, and water from hot springs (2–8). Recently, *Paracoccus versutus* MAL 1HM19, isolated from the Mae Um Long Luang hot spring (Thailand), was reported for its unique ability to simultaneously convert H_2_S and NO_3_^−^ to sulfate (SO_4_^2–^) and dinitrogen (N_2_) via the H_2_S oxidation and NO_3_^−^ reduction pathways (8), respectively.

The *P. versutus* strain MAL 1HM19 was cultured in modified Coleville synthetic brine (mCSB) under the following conditions: temperature, 35°C; pH 7.0; and incubation time, 72 h. Then, the genomic DNA of *P. versutus* MAL 1HM19 was extracted using the DNeasy PowerSoil pro kit (Qiagen, Germany). The quality and quantity of the DNA were tested, and the DNA was sequenced using an Illumina HiSeq 4000 instrument by CD Genomics (NY, USA), according to Mira et al. (9). As a first step, the purified DNA samples were sheared into smaller fragments with the desired sizes using an E210 ultrasonicator (Covaris, Inc., USA). Thereafter, DNA fragments of ∼500 bp were selected to prepare the library for paired-end (PE) sequencing. The specific oligonucleotides (80-mer Illumina adapters) were ligated with both ends of the DNA fragments using the TA Cloning method. Finally, the qualified library was submitted for sequencing using an Illumina HiSeq 4000 instrument with a 2 × 150-bp PE sequencing configuration. The generated data were used for the downstream bioinformatics analysis. Raw reads (4,335,411 reads) generated on the sequencing platform were filtered and checked for quality using the DynamicTrim and LengthSort Perl scripts in the SolexaQA suite (9). The clean reads (3,863,478 reads) were mapped with the reference genome (*P. versutus* DSM 582) using the Burrows-Wheeler Aligner (BWA) software (10). The Q20 and Q30 statistics of the clean reads in all samples exceeded 93.41% and 85.18%, respectively. Clean data were assembled using SOAP*denovo* version 2.04 (11). For all software programs used in this study, the default parameters were used, unless otherwise noted. The genome was annotated based on the NCBI Prokaryotic Genome Annotation Pipeline (12).

The genome of *P. versutus* MAL 1HM19 was composed of 5,427,451 bp, with a 67.60% GC content distributed in 245 contigs, providing 207× average genome coverage; 88.76% of the genome was covered, and 5,204 candidate genes were found in this microorganism. Among the 5,464 predicted genes, 5,404 genes were identified as protein-coding genes (CDSs), while 3.7% of the total genes were predicted to be pseudogenes. Moreover, the genome also contained 53 RNA genes (5S, 16S, and 23S rRNAs) and 54 tRNA genes. The *N*_50_ and *L*_50_ values were 65,835 bp and 26, respectively, while the largest contig from the draft genome sequence was 279,639 bp long.

### Data availability.

The draft genome sequence of *P. versutus* MAL 1HM19 has been deposited in GenBank under the accession number QPML00000000 and BioProject number PRJNA482279. The raw sequencing reads have been deposited in the NCBI Sequence Read Archive (SRA) under the accession number SRR10902179.

## References

[1] Pokorna D, Zabranska J 2015 Sulfur-oxidizing bacteria in environmental technology. Biotechnol Adv 33:1246–1259. doi:10.1016/j.biotechadv.2015.02.007. 25701621

[2] Li K, Wang S, Shi Y, Qu J, Zhai Y, Xu L, Xu Y, Song J, Liu L, Rahman MA, Yan Y 2011 Genome sequence of *Paracoccus* sp. strain TRP, a chlorpyrifos biodegrader. J Bacteriol 193:1786–1787. doi:10.1128/JB.00014-11. 21257769 PMC3067656

[3] Kumaraswamy R, Sjollema K, Kuenen G, Van Loosdrecht M, Muyzer G 2006 Nitrate-dependent [Fe(II)EDTA]^2−^ oxidation by *Paracoccus ferrooxidan*s sp. nov., isolated from a denitrifying bioreactor. Syst Appl Microbiol 29:276–286. doi:10.1016/j.syapm.2005.08.001. 16682296

[4] Siller H, Rainey FA, Stackebrandt E, Winter J 1996 Isolation and characterization of a new Gram-negative, acetone-degrading, nitrate-reducing bacterium from soil, *Paracoccus solventivorans* sp. nov. Int J Syst Bacteriol 46:1125–1130. doi:10.1099/00207713-46-4-1125. 8863446

[5] Qiao L, Wang JL 2010 Microbial degradation of pyridine by *Paracoccus* sp. isolated from contaminated soil. J Hazard Mater 176:220–225. doi:10.1016/j.jhazmat.2009.11.016. 19945787

[6] Wang J, Jiang X, Liu X, Sun X, Han W, Li J, Wang L, Shen J 2018 Microbial degradation mechanism of pyridine by *Paracoccus* sp. NJUST30 newly isolated from aerobic granules. Chem Eng J 344:86–94. doi:10.1016/j.cej.2018.03.059.

[7] Lin D, Zhu S, Chen Y, Huang Y, Yang J, Chen J 2019 *Paracoccus indicus* sp. nov., isolated from surface seawater in the Indian Ocean. Antonie Van Leeuwenhoek 112:927–933. doi:10.1007/s10482-019-01226-2. 30737708

[8] Watsuntorn W, Ruangchainikom C, Rene ER, Lens PN, Chulalaksananukul W 2017 Hydrogen sulfide oxidation under anoxic conditions by a nitrate-reducing, sulfide-oxidizing bacterium isolated from the Mae Um Long Luang hot spring, Thailand. Int Biodeterior Biodegradation 124:196–205. doi:10.1016/j.ibiod.2017.06.013.

[9] Mira NP, Münsterkötter M, Dias-Valada F, Santos J, Palma M, Roque FC, Guerreiro JF, Rodrigues F, Sousa MJ, Leão C, Güldener U, Sá-Correia I 2014 The genome sequence of the highly acetic acid-tolerant *Zygosaccharomyces bailii*-derived interspecies hybrid strain ISA1307, isolated from a sparkling wine plant. DNA Res 21:299–313. doi:10.1093/dnares/dst058. 24453040 PMC4060950

[10] Li H, Durbin R 2010 Fast and accurate long-read alignment with Burrows-Wheeler transform. Bioinformatics 26:589–595. doi:10.1093/bioinformatics/btp698. 20080505 PMC2828108

[11] Li R, Zhu H, Ruan J, Qian W, Fang X, Shi Z, Li Y, Li S, Shan G, Kristiansen K, Li S, Yang H, Wang J, Wang J 2010 *De novo* assembly of human genomes with massively parallel short read sequencing. Genome Res 20:265–272. doi:10.1101/gr.097261.109. 20019144 PMC2813482

[12] Tatusova T, DiCuccio M, Badretdin A, Chetvernin V, Nawrocki EP, Zaslavsky L, Lomsadze A, Pruitt KD, Borodovsky M, Ostell J 2016 NCBI Prokaryotic Genome Annotation Pipeline. Nucleic Acids Res 44:6614–6624. doi:10.1093/nar/gkw569. 27342282 PMC5001611

